# Soft molecularly imprinted nanoparticles with simultaneous lossy mode and surface plasmon multi-resonances for femtomolar sensing of serum transferrin protein

**DOI:** 10.1038/s41598-023-38262-y

**Published:** 2023-07-11

**Authors:** Francesco Arcadio, Laurent Noël, Domenico Del Prete, Devid Maniglio, Mimimorena Seggio, Olivier Soppera, Nunzio Cennamo, Alessandra Maria Bossi, Luigi Zeni

**Affiliations:** 1 Department of Engineering, University of Campania “L. Vanvitelli”, Via Roma 29, Aversa, Italy; 2 CNRS, IS2M UMR 7361, University of Upper-Alsace, 68100 Mulhouse, France; 3 Université de Strasbourg, 67000 Strasbourg, France; 4 Department of Industrial Engineering, University of Trento, Via Sommarive 34, 38123 Trento, Italy; 5 Department of Biotechnology, University of Verona, Strada Le Grazie 15, 37134 Verona, Italy

**Keywords:** Sensors, Nanoparticles, Biosensors

## Abstract

The simultaneous interrogation of both lossy mode (LMR) and surface plasmon (SPR) resonances was herein exploited for the first time to devise a sensor in combination with soft molecularly imprinting of nanoparticles (nanoMIPs), specifically entailed of the selectivity towards the protein biomarker human serum transferrin (HTR). Two distinct metal-oxide bilayers, i.e. TiO_2_–ZrO_2_ and ZrO_2_–TiO_2_, were used in the SPR–LMR sensing platforms. The responses to binding of the target protein HTR of both sensing configurations (TiO_2_–ZrO_2_–Au-nanoMIPs, ZrO_2_–TiO_2_–Au-nanoMIPs) showed femtomolar HTR detection, LODs of tens of fM and K_Dapp_ ~ 30 fM. Selectivity for HTR was demonstrated. The SPR interrogation was more efficient for the ZrO_2_–TiO_2_–Au-nanoMIPs configuration (sensitivity at low concentrations, S = 0.108 nm/fM) than for the TiO_2_–ZrO_2_–Au-nanoMIPs one (S = 0.061 nm/fM); while LMR was more efficient for TiO_2_–ZrO_2_–Au-nanoMIPs (S = 0.396 nm/fM) than for ZrO_2_–TiO_2_–Au-nanoMIPs (S = 0.177 nm/fM). The simultaneous resonance monitoring is advantageous for point of care determinations, both in terms of measurement’s redundancy, that enables the cross-control of the measure and the optimization of the detection, by exploiting the individual characteristics of each resonance.

## Introduction

Optical sensors provide analytical solutions to the most diverse needs, ranging from environmental monitoring to industrial processes and biomedical applications. At the foundation of this widespread diffusion, are the inherent and remarkable advantages offered by the optical sensing technology: the label-free and real-time detection, the passive nature, the immunity to electromagnetic interferences, the possibility of miniaturization and of in parallel array-monitoring^1–3^. Moreover, optical sensing can rely on a range of physical phenomena, including absorption, fluorescence, plasmonics, fuelling the diversification in the final configurations of the optical sensing device.

Plasmonic is among the most exploited optical phenomena, enabling label-free, real-time sensing of down-to quasi-single molecule, in vivo and in situ^4–6^. In fact, perturbation of the surface plasmon resonance conditions can occur even for few binding events (femtomolar) happening at the interface between the metal and the dielectric. In particular, surface plasmon resonance (SPR) gained foremost importance in sensing. In SPR, electromagnetically induced coherent delocalized electron oscillations at the interface between a metal and a dielectric allow to monitor modifications of the local refractive index (RI), providing insights to the molecular events at the interface^6,7^. The SPR phenomenon coupled to optical fibre sensors, enables a wide variety of applications, encompassing gas sensing^8,9^, biochemical field sensing^10–13^, magnetic field detection, etc.^14–16^. Yet, SPR requires a high index over surface to shift the operating point towards aqueous environment and this reduces the sensitivity^17^.

Lately, lossy mode resonance (LMR), that is a resonance phenomenon conditional to waveguides coated with thin films of optical absorbing materials, or lossy materials, was exploited for fiber optic sensing. Initially, LMR was described as the propagation of light in semiconductor cladded waveguides that experienced some attenuation maxima for specific thickness values of the semiconductor cladding and, also, at certain wavelengths of incidence values^18^. This was due to a coupling between waveguide modes and a specific lossy mode of the semiconductor thin film^2,19–21^. The LMR phenomenon is not limited to semiconductor claddings, instead it can be observed for dielectric claddings^22^, including polymers^23^. In fact, LMRs occur when the real part of the thin-film permittivity is positive and higher in magnitude than both its own imaginary part and the real part permittivity of the materials surrounding the thin-film (waveguide and external medium as well). Advantages of LMRs are the possibility to fine-tune the spectral position just by changing the thickness of the lossy coating; the possibility to rely on several resonances, that can appear when the thickness of the lossy coating is increased, hence any peak can be exploited for sensing. Due to these characteristics LMR-based optical fiber sensors are increasingly described in the literature^21,22,24–26^.

Worth of note is the possibility to take advantage of both SPR and LMR and few configurations able to simultaneously excite both phenomena are also reported^27–29^. As an example, da Silva et al.^29^ developed a RI and corrosion sensor which consisted of two cascaded D-shaped sensor regions: the first one exploiting the LMR of a bilayer of titanium dioxide–aluminum and an SPR sensitive area consisting of a bilayer of gold and titanium dioxide. Along this line, Cennamo et al. have recently described an experimental configuration able to trigger both SPR and LMR phenomena on a single sensor platform^30^. To this aim, a plasmonic sensor based on plastic optical fiber (POF)^31^ was modified by depositing a combination of metal oxides (ZrO_2_ and TiO_2_) as an intermediate layer between the exposed fiber core and the thin gold film^30^. In such a way, by exploiting several kinds of metal-oxides configurations, the platform exploited both SPR and LMR resonances to measure RIs at the metal interface^30^.

In the present work, the SPR–LMR-POF platform has been used for the first time in the biosensing field, by functionalizing the gold surface on the top of the metal oxides bilayer with molecularly imprinted polymers, synthesized in form of soft imprinted nanogels (nanoMIPs) and specific for the detection of the serum marker protein human transferrin (HTR). The nanoMIPs were characterized by hydrodynamic size of about 100 nm, high surface to volume ratio, fast mass transfer kinetics, and a limited number of binding sites per nanoparticle^32,33^.

Two SPR–LMR configurations functionalized with nanoMIPs and based either on the metal-oxides bilayer TiO_2_–ZrO_2_ or on the ZrO_2_–TiO_2_ were experimentally tested for their binding behaviour and for the selectivity. The comparative analysis of the sensing capabilities offered by TiO_2_–ZrO_2_ or ZrO_2_–TiO_2_ platforms, highlighted pros and cons of both experimental configurations.

## Results

Recently, we described the optical behaviour of both a ZrO_2_–TiO_2_–Au and a TiO_2_–ZrO_2_–Au D-shaped POF configurations^30^, characterized by an SPR minimum at about 600 nm and a LMR maximum at about 500 nm. It was observed that the SPR and LMR resonance wavelengths of both configurations, when challenged with water-glycerine solutions of increasing refractive indexes (RIs), ranging from 1.332 (water) to 1.385, increased (red shift). These results are reported in Figures S2 and S3 (Supplementary Information), respectively.

### Functionalization of the SPR–LMR POF probes with nanoMIPs

**Figure 1:**
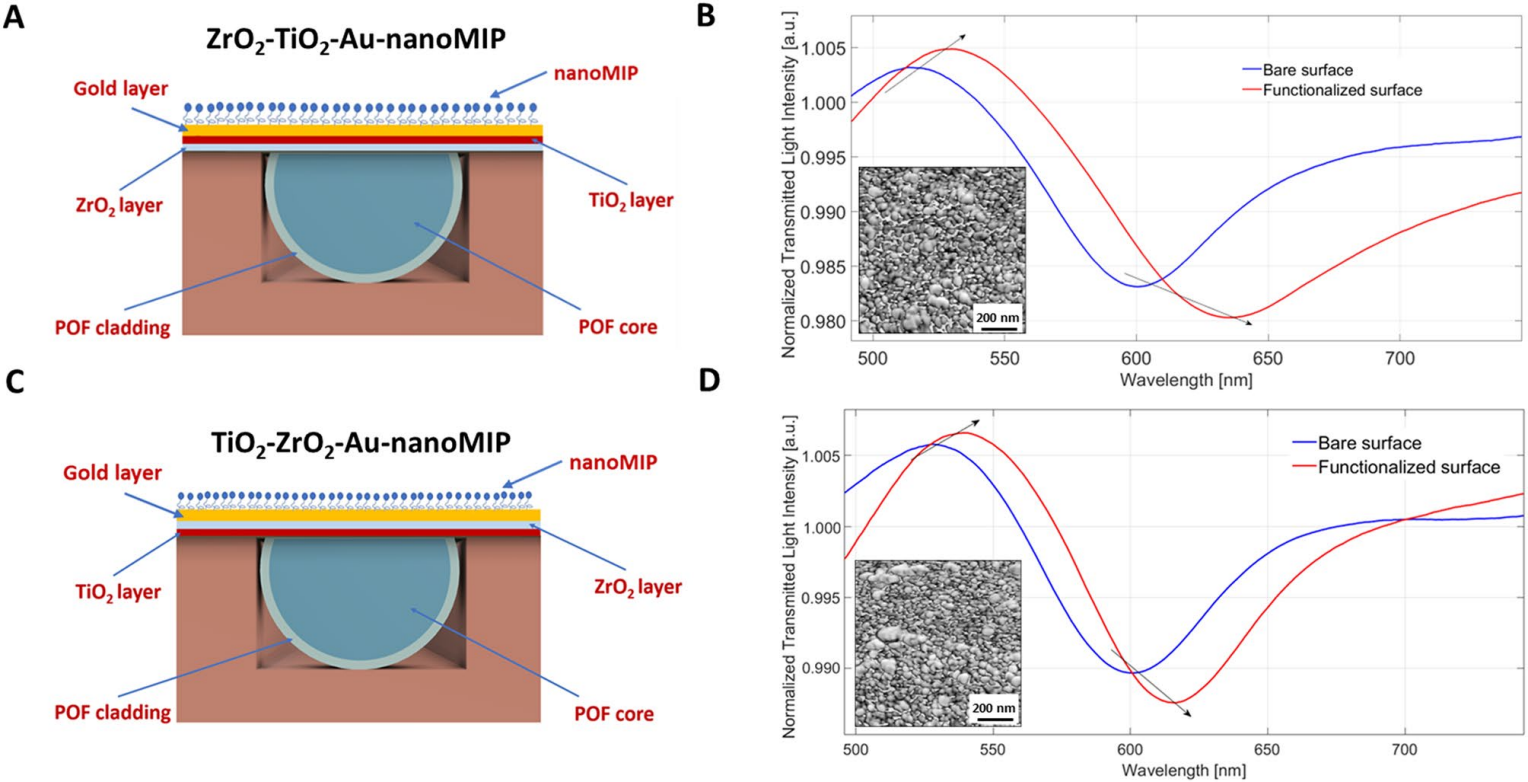
Cross-sections of (**A**) ZrO_2_–TiO_2_–Au and (**C**) TiO_2_–ZrO_2_–Au configuration platforms after the functionalization process with nanoMIP. Normalized transmitted spectra for (**B**) ZrO_2_–TiO_2_–Au and (**D**) TiO_2_–ZrO_2_–Au configurations, acquired with water as the surrounding medium, obtained before (blue line) and after (red line) the functionalization process. Spectra were normalized for the reference spectrum, that was acquired in air. The functionalization-dependent red-shifts in the SPR in the region 600–650 nm and in the LMR at 500 nm are marked by the arrows. The inset in (**B**) and (**D**) shows the surface topography of the ZrO_2_–TiO_2_–Au and TiO_2_–ZrO_2_–Au nanoMIP’s functionalized sensing area imaged by atomic force microscopy.

Both the ZrO_2_–TiO_2_–Au and the TiO_2_–ZrO_2_–Au probes were functionalized with nanoMIPs. Figure 1A and C show an outline of the sections of the two configurations modified with the nanoMIP layer. The effective functionalization of both platforms with nanoMIPs was optically evaluated. The normalized spectra before and after the functionalization process were compared (Fig. 1B and D). Both the SPR and LMR resonance wavelengths exhibited a red shift, confirming a successful nanoMIP layer deposition on the gold surface (Fig. 1B and D). Moreover, the functionalization of the probes with the nanoMIPs was also controlled by atomic force microscopy (inset in Fig. 1B and D), further confirming the effective nanoMIP coupling.

### Analysis of the resonances of nanoMIP’s functionalized probes with water-glycerine solutions

**Figure 2:**
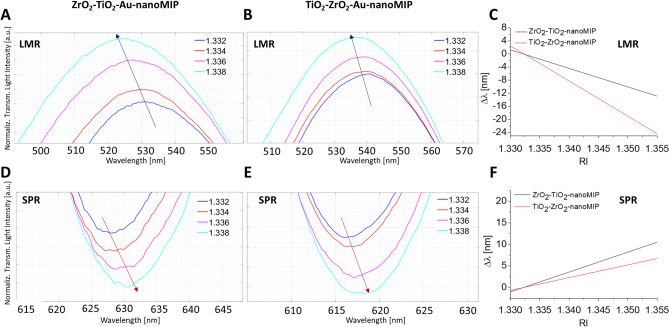
Resonance wavelength analysis of nanoMIP’s functionalized probes with water-glycerine solutions. Enlargement of LMR (**A**) for ZrO_2_–TiO_2_–Au-nanoMIP and (**B**) TiO_2_–ZrO_2_–Au-nanoMIP and enlargement of SPR (**D**) for ZrO_2_–TiO_2_–Au-nanoMIP and (**E**) TiO_2_–ZrO_2_–Au-nanoMIP resonance wavelength areas of normalized spectra obtained for different external RIs. Whole spectra are reported in Figure S4 and S5 (Supplementary Information). Sensor’s responses in terms of resonance wavelength variation (Δλ) versus external RI for (**C**) LMR and (**F**) SPR.

Both the ZrO_2_–TiO_2_–Au-nanoMIP and the TiO_2_–ZrO_2_–Au-nanoMIP experimental configurations were optically characterized by using water-glycerin mixtures. The spectra, presented in Fig. 2, showed the red shifting of the SPR resonance for increasing external RIs, alike what reported for bare probes^30^. Interestingly, solutions at increasing RIs caused the blue-shift of the LMR resonance wavelengths, in contrast to what observed on bare surfaces, prior to the nanoMIP functionalization (see Figures S2 and S3). The LMR resonance reverted from a red-shift in the absence of nanoMIPs to a blue-shift after the nanoMIPs conjugation, demonstrating that the nanoMIP layer greatly influenced the LMR phenomenon both for the ZrO_2_–TiO_2_–Au-nanoMIP and the TiO_2_–ZrO_2_–Au-nanoMIP configurations. It was reported in the literature that the characteristics of LMR resonance are strongly determined by the nanometric film deposited on the sensing area. Any change in the nanocoating parameters affect the characteristics of LMR, due to the effective RI resulting from the modified coupled modes^34,35^.

### Binding tests of SPR–LMR POF ZrO_2_–TiO_2_–Au-nanoMIP versus TiO_2_–ZrO_2_–Au-nanoMIP probes

**Figure 3:**
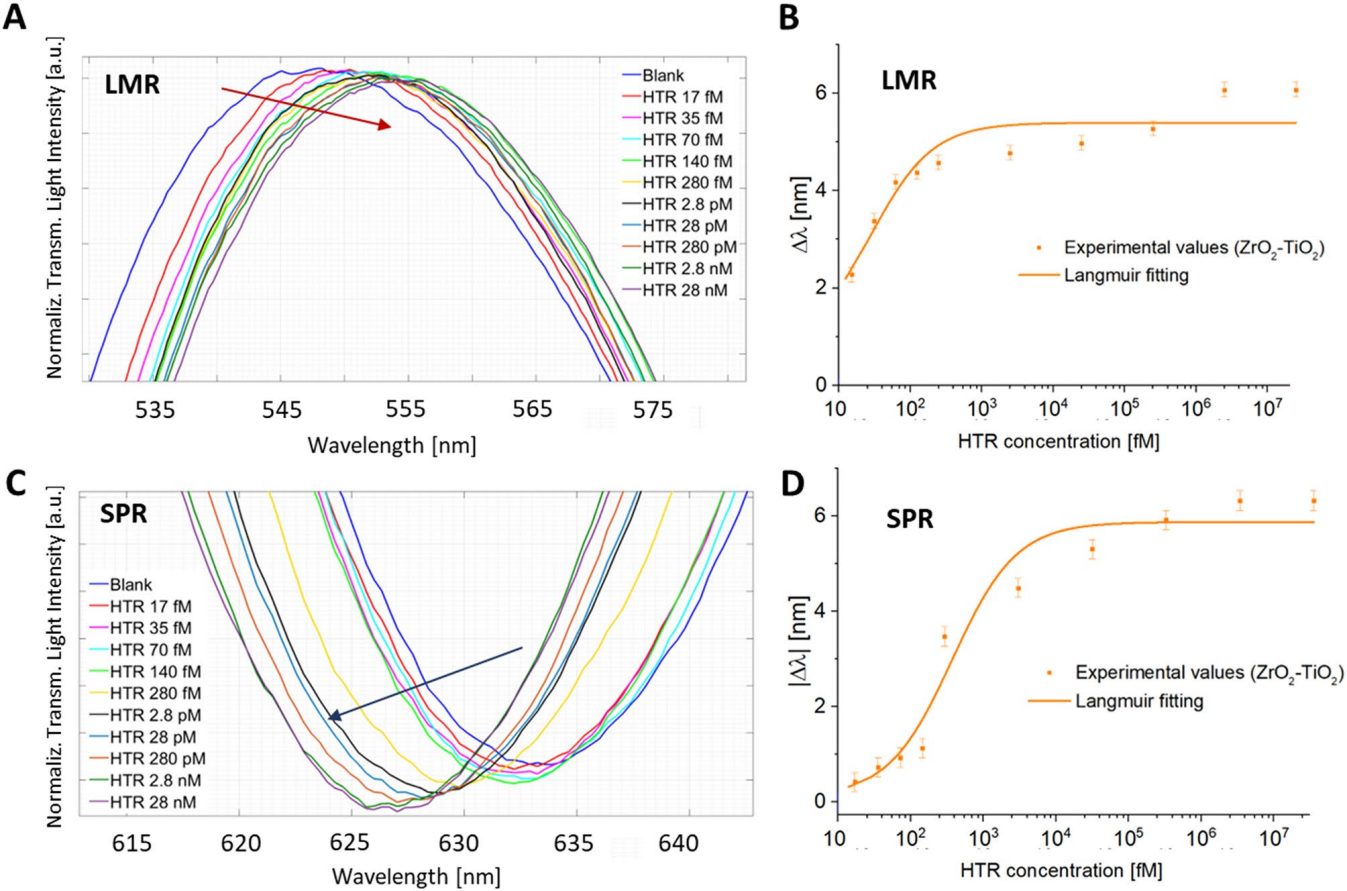
ZrO_2_–TiO_2_–Au-nanoMIP configuration: enlargement of the (**A**) LMR resonance wavelength, and (**C**) SPR resonance wavelength areas. The LMR spectra of Fig. 3A were shifted in the y-axis direction for clarity. Binding curves and Langmuir fitting for the response of ZrO_2_–TiO_2_–Au-nanoMIP configuration to the HTR binding: resonance wavelength variations, calculated with respect to the blank, versus different concentrations of HTR for (**B**) LMR and (**D**) SPR peaks (in absolute value). Error bars from n = 3. The error bars were calculated as the maximum measured variation in resonance wavelengths and were found to be 0.2 nm. Whole spectra are reported in Figure S6 (Supplementary Information).

**Figure 4:**
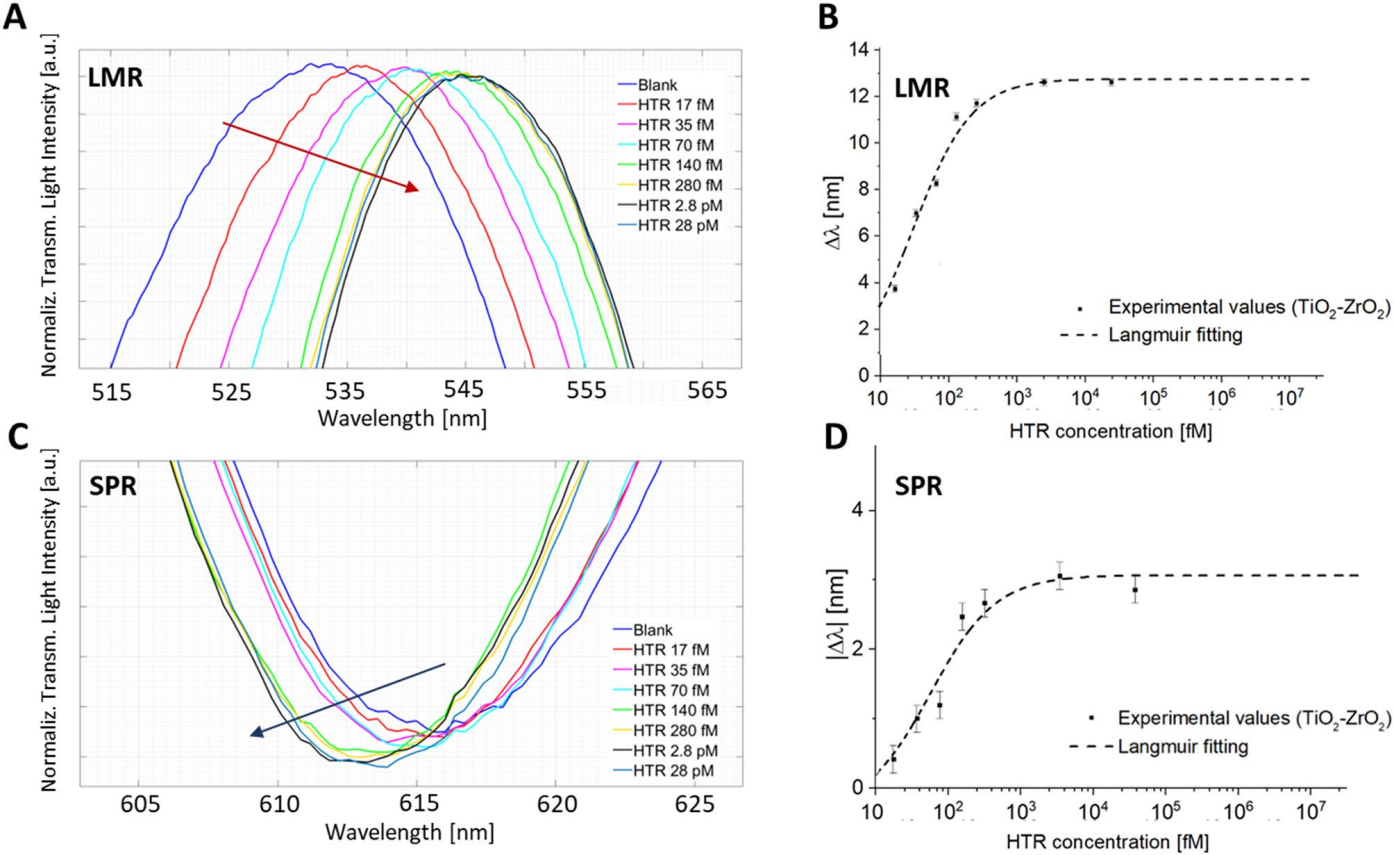
TiO_2_–ZrO_2_–Au-nanoMIP configuration: enlargement of the (**A**) LMR resonance wavelength, and (**C**) SPR resonance wavelength areas. The LMR spectra of Fig. 4A were shifted in the y-axis direction for clarity. Binding curves and Langmuir fitting for the response of TiO_2_–ZrO_2_–Au-nanoMIP configuration to the HTR binding: resonance wavelength variations, calculated with respect to the blank, versus different concentrations of HTR for (**B**) LMR and (**D**) SPR peaks (in absolute value). Error bars from n = 3.The error bars were calculated as the maximum measured variation in resonance wavelengths and were found to be 0.2 nm. Whole spectra are reported in Figure S7 (Supplementary Information).

Next, the binding of the target analyte, the protein HTR, onto ZrO_2_–TiO_2_–Au-nanoMIP and TiO_2_–ZrO_2_–Au-nanoMIP configurations was studied. Each sensing platform was challenged with increasing HTR concentrations, ranging from 17 fM to 28 nM and the LMR and SPR resonances were acquired. The behaviour of ZrO_2_–TiO_2_–Au-nanoMIP configuration at binding was reported in Fig. 3A and C for LMR and SPR respectively. A blueshift was observed for the SPR resonance upon binding of HTR (Fig. 3C), in accordance with the literature, in which nanoMIPs, that are soft nanogels, have been reported to deform and shrink upon interacting with the target analyte, causing a blue-shift in the SPR^36^. In contrast, the binding effect on the LMR resonance resulted in a red-shift (Fig. 2A), in agreement with earlier observations^34^. From the comparison of Fig. 3A and C it appeared that the SPR and LMR resonance phenomena at binding behaved inversely. The LMR and SPR isotherms describing the binding of HTR to the nanoMIP receptor’s layer on the ZrO_2_–TiO_2_–Au platform were respectively shown in Fig. 3B and D, along with the Langmuir fitting of the experimental data and the specific fitting parameter were reported in Table S1 (Supplementary Information). Concerning the TiO_2_–ZrO_2_–Au-nanoMIP platforms, the binding behaviour was reported in Fig. 4. The LMR (Fig. 4A) and SPR (Fig. 4C) showed the normalized transmitted spectra for increasing HTR concentrations (17 fM to 28 pM). In similarity to what observed for the ZrO_2_–TiO_2_–Au-nanoMIP configuration, the binding response of the SPR resonance was opposite to the LMR resonance.

Indeed, Fig. 4A and C, showed that the binding of increasing concentrations of HTR blue shifted the SPR resonance wavelength, whereas the LMR one increased it (red shift). Figure 4B and D provided the binding isotherms for the interaction of HTR to the TiO_2_–ZrO_2_–Au-nanoMIP configuration, for the SPR (Fig. 4B) and LMR (Fig. 4D) resonances, along with the Langmuir fittings of the experimental data. Table S2 (Supplementary Information) reported the parameter related to the Langmuir curves used to fit the experimental values.

### Selectivity tests

**Figure 5:**
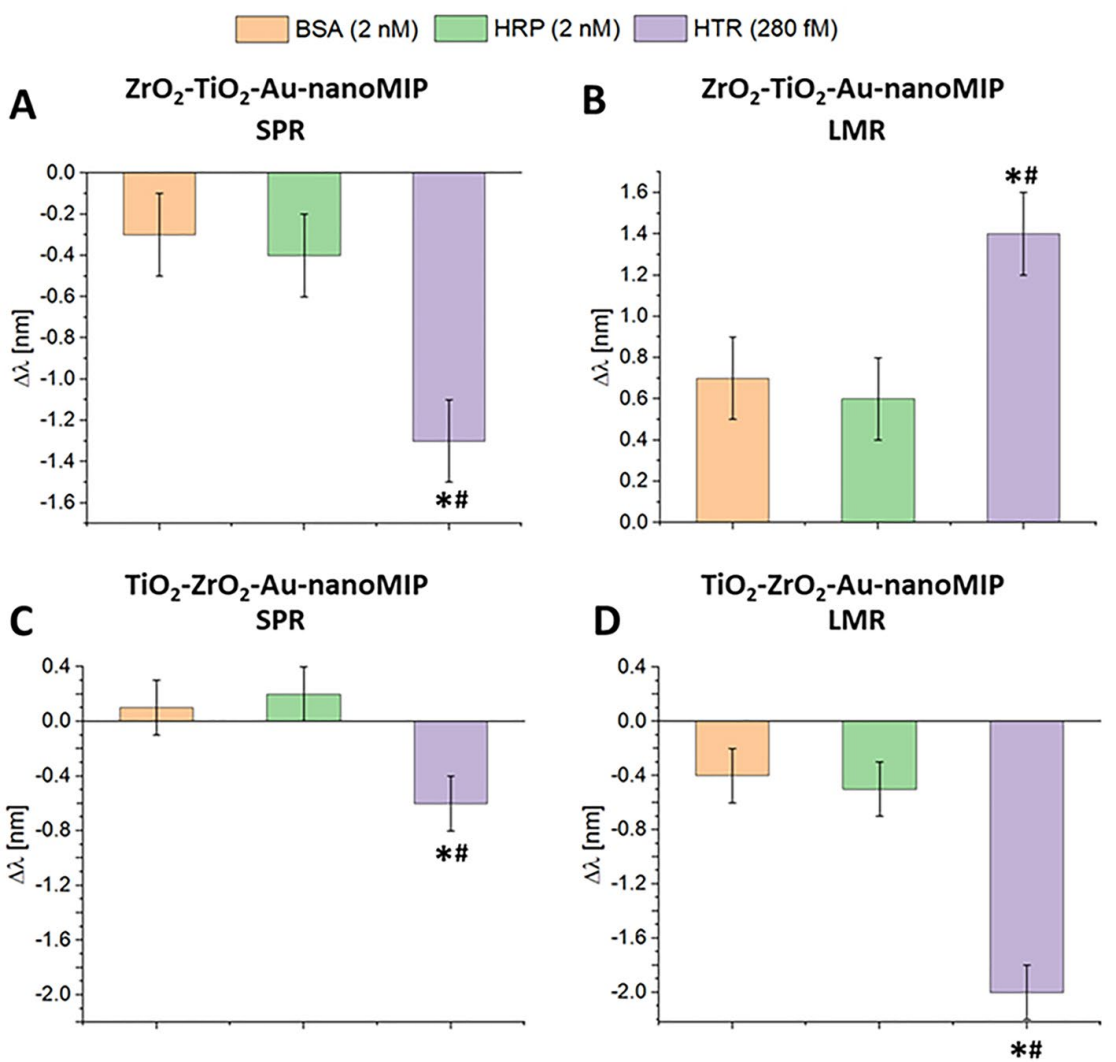
Selectivity test for the proposed configurations for different proteins: resonance wavelength variation with respect to blank for (**A**) SPR and (**B**) LMR resonances relative to ZrO_2_–TiO_2_-Au-nanoMIP configuration and (**C**) SPR and (**D**) LMR resonances relative to TiO_2_–ZrO_2_–Au-nanoMIP configuration. One-way ANOVA *p < 0.05 versus BSA and #*p* < 0.05 versus HRP. The error bars were calculated as standard deviation of the dataset (n = 3, St.Dev. = 0.2 nm).

In order to confirm the specific binding of the nanoMIPs conjugated to the SPR–LMR POF platforms, selectivity tests were carried out by comparing the shifts in resonance wavelength of the target analyte (HTR) with two non-related protein interferents, namely bovine serum albumin (BSA) and horseradish peroxidase (HRP), both at the concentration of 2 nM. HTR is a protein of about 75,000 g/mol in molecular weight and with a neutral isoelectric point (pI). BSA was chosen for the similarity, in terms of aminoacid composition, pI and molecular weight (66.000 g/mol) to human serum albumin, which is the most abundant protein in human serum, hence it is a prominent competitor of HTR in serum samples. As an alternative non-template protein, HRP was chosen, because it is a monomer of 44,000 g/mol that can dimerize 88,000 g/mol, so results were aimed at understanding the effect of size on the recognition. Concerning the ZrO_2_–TiO_2_–Au-nanoMIP configuration, Fig. 5A and B reported, respectively for SPR and LMR phenomena, the wavelength shifts for the interferents compared to the shift obtained for HTR (280 fM). The shifts caused by the interferents were negligible respect to the effect of the analyte at a much lower concentration, fully supporting the selectivity of the nanoMIPs. In a similar manner, the selectivity of the TiO_2_–ZrO_2_–Au-nanoMIP configuration was also tested (Fig. 5C and D), confirming the shifts caused by the interferents were negligible respect to the ones caused by the analyte at a much lower concentration.

## Discussion

The present study was intended to investigate the optical behaviour of ZrO_2_–TiO_2_–Au-nanoMIP versus TiO_2_–ZrO_2_–Au-nanoMIP interfaces, with the aim of contributing to elucidate the role and the potential of multi-interrogation based on both LMR and SPR on sensing platforms where the receptor layer consisted of soft, deformable molecularly imprinted nanoparticles.

The optical behaviours of both kinds of platforms, prior and after the nanoMIP functionalization, is summarized in Table 1. It was observed that the SPR signal of both TiO_2_–ZrO_2_–Au-nanoMIPs and ZrO_2_–TiO_2_–Au-nanoMIPs red shifted for increasing RIs of the surrounding medium. In contrast, the LMR phenomenon appeared to be oppositely influenced by the nanoMIP layer, such that, for increasing RIs, changes in the boundary conditions at the resonance, reversed the resonance shifts from red to blue. Such an effect finds ground in the sensitivity of the LMR phenomenon to the surroundings, as mathematically demonstrated by Zhao and Wang^37^. Concerning the sensing performance of the nanoMIP-functionalized metal oxides bilayers platforms, the binding of the targeted analyte (HTR) produced a blue-shift of the SPR, while a red-shift of the LMR resonances. The blue-shift of the SPR resonance was consistent with binding measurements previously observed with a nanoMIP functionalized D-shaped POF^36^, in which the binding of the target analyte led to the deformation of the soft nanoMIPs, resulting in an apparent decrease of the RIs at the surface. In general, the blueshifts at binding is a condition described in the case of analytes interacting with gel-type of receptor-layers, such as the case of soft MIP and nanoMIP materials, where changes in the water content of the gel produce significant deformations at the sensing surface^38^.

**Table 1:** Resonance wavelength shifts at the increasing of RI for the studied configurations.

Multilayer type on the D-shaped POF	Kind of resonance wavelength shift measured at the increasing of RI	
	SPR	LMR
ZrO_2_–TiO_2_–Au	Red-shift	Red-shift
TiO_2_–ZrO_2_–Au	Red-shift	Red-shift
ZrO_2_–TiO_2_–Au-nanoMIP	Red-shift	Blue-shift
TiO_2_–ZrO_2_–Au-nanoMIP	Red-shift	Blue-shift

Concerning the changes in the LMR resonance upon HTR binding, these were observed to red-shift. It was expected that the functionalization of the gold-coated metal oxide bilayers with the nanoMIPs, modifies the interface layer characteristics, affecting the LMR resonance behavior^34,35^. In the present case, the LMR shifts observed at binding were opposite to the SPR. Such opposite behavior resulted in the red shift of the LMR wavelength and appeared coherent with the optical characterization shown in Table 1.

In fact, the LMR showed to blueshift for increasing RIs of the surrounding medium, thus in analogy to SPR, it was expected behave oppositely at binding, hence producing a red-shift upon the apparent decreasing of RIs. Given that, the binding of the analyte to the soft nanoMIP was previously observed to produce an apparent decrease of the RIs at the sensing interface, the herein observed LMR shifts were in accordance to such apparent RIs decrease. A comparison between the binding behaviour of the two configurations, both considering LMR and SPR, was performed and results are summarized in Table 2. Quantitative comparative parameters for the two configurations were gained from Eq. (1). The comparative analysis presented in Table 2 indicated that, with respect to the SPR resonance, the ZrO_2_–TiO_2_–Au-nanoMIP had a greater sensitivity at low concentrations and a lower LOD than the TiO_2_–ZrO_2_–Au-nanoMIP configuration. Conversely, for the LMR resonance, TiO_2_–ZrO_2_–Au-nanoMIP had a lower LOD, but a greater sensitivity at low concentrations. It is worth noting that the total wavelength shift of the SPR is greater for the ZrO_2_–TiO_2_–Au-nanoMIP configuration, while the opposite is observed for the LMR. These findings are consistent with the results obtained in the optical characterization presented in Cennamo et al.^30^ which demonstrated that the ZrO_2_–TiO_2_–Au configuration exhibits the best SPR bulk sensitivity, while the TiO_2_–ZrO_2_ configuration shows the best LMR bulk sensitivity.

**Table 2:** Analytical parameters for a quantitative assessment of the binding behaviour for the ZrO_2_–TiO_2_–Au-nanoMIP and TiO_2_–ZrO_2_–Au-nanoMIP configurations.

Configuration	Resonance	${\mathrm{S}}_{\mathrm{lowc}}$* [nm/fM]	$\mathrm{LOD}$** [fM]	${\mathrm{K}}_{\mathrm{aff}}$*** [fM-1]
ZrO_2_–TiO_2_–Au-nanoMIP	SPR	0.108	13.6	0.018
	LMR	0.177	12.9	0.032
TiO_2_–ZrO_2_–Au-nanoMIP	SPR	0.061	16.1	0.019
	LMR	0.396	9.5	0.031

*Sensitivity at low concentration, S_lowc_, refers to the slope of the curve (Eq. 1) when the analyte concentrations satisfy the condition of c much lower than K, that can be considered linear $\left(\left|{\Delta {\varvec{\uplambda}}}_{\mathbf{m}\mathbf{a}\mathbf{x}}\right|/\mathbf{K}\right)$; **Limit of detection (LOD) was calculated as the ratio between three times the standard deviation of the blank and the sensitivity at low concentration $\left((3\times \mathbf{S}\mathbf{t}.\mathbf{d}\mathbf{e}\mathbf{v}.\mathbf{o}\mathbf{f}{{\varvec{\uplambda}}}_{0})/{\mathbf{S}}_{\mathbf{l}\mathbf{o}\mathbf{w}\mathbf{c}}\right)$. ***Affinity constant was **1/K** of Eq. 1.

Finally, a comparative analysis was conducted between the best configurations described in this study and literature data reporting about other sensors, functionalized with MIPs and other kinds of receptors and targeting analytes with molecular weights similar to the HTR used in this study. Specifically, the sensors were compared in terms of their limit of detection (LOD), and the results are presented in Table 3.

**Table 3:** Comparison between the best configurations presented in this work and other sensor already presented in literature.

Sensor	Resonance	Receptor	Analyte	LOD	References
SPR-LDF sensor	SPR	nanoMIP	HTR	4.3 fM	^10^
SPR-sensor chip	SPR	boronic acid	HTR	4.4 nM	^39^
Planar waveguide	LMR	antibody	anti-IgG	2.2 µg/mL*	^35^
Optical fiber sensor	LMR	aptamer	CRP	0.062 mg/L**	^40^
CQD@SiO_2_-MIP	LMR	MIP	Epinephrine	0.72 mM	^41^
SiO_2_-NH_2_@GO-MIP-PDA	LMR	MIP-PDA	Epinephrine	70 nM	^42^
ZnO/MoS_2_-MIP	LMR	MIP	p-cresol	28 nM	^43^
ZrO_2_–TiO_2_–Au-nanoMIP	SPR	nanoMIP	HTR	13.6 fM	This work
TiO_2_–ZrO_2_–Au-nanoMIP	LMR	nanoMIP	HTR	9.5 fM	This work

*LOD corresponded to 10 nM; **LOD corresponded to 6.2 nM.

Table 3 showed that a sensitive interrogation, such as LMR or SPR, enable to detect binding events in a broad range of concentrations, with LODs depending on the kind of chosen receptor. Non-deformable, rigid MIP surfaces, of hundreds of nanometer thickness, such as in the case plastic optical fiber (POF) modified with silica-based MIPs with embedded carbon dots, did report a LOD of 0.72 mM^41^. In the case of a multimode POF modified with an aminosilane and graphene oxide onto which a nanolayer of polydopamine MIP (~ 70 nm) was deposited, the LOD for the target analyte was 70 nM^42^. Finally, an uncladded multimode silica fiber modified with ZnO/MoS_2_ onto which a thin layer of methacrylate MIP was thermally synthesized, did report a LOD of 28 nM and a remarkable peak shift, when interrogated in LMR^43^. Worth to note is that the femtomolar LODs were achieved just when soft nanoMIPs, which undergo to a peculiar deformation at binding, are chosen as receptor element, as already reported in SPR^36^.

## Conclusions

The simultaneous generation of LMR and SPR resonances on a same planar platform has been reported by Fuentes et al.^27^ and further Gaur and colleagues^44^ investigated the interplay of these optical phenomena and the dependence of one to another from a theoretical and practical point of view, by studying bilayers of ITO and Ag and trilayers ITO-Ag-ITO, exploring different thicknesses and reporting how the sensitivity varies in the different layer configurations^44^. It resulted that depending on the layer thicknesses one resonance dominates the other^44^. In the present work, it was demonstrated that a POF modified with bilayers of ZrO_2_–TiO_2_ or TiO_2_–ZrO_2_ and followed by a gold layer^30^, provided LMR and SPR and can be used in combination with highly selective, soft nanoMIPs to develop a new, cheap and ultralow sensitive sensing platform.

It was observed that, the LMR phenomenon was strongly influenced by the nanoMIP layer, whose effect was to modify the boundary conditions at the resonance, reversing the type of resonance from red to blue shift. Concerning the SPR resonance, the best configuration was the one based on the ZrO_2_–TiO_2_ bilayer, that had a sensitivity at low concentration of about 0.108 nm/fM with an LOD of 13.6 fM. On the opposite, concerning the LMR resonance, the configuration based on the TiO_2_–ZrO_2_ bilayer resulted the best one, showing a sensitivity at low concentration of 0.396 nm/fM and an LOD of 9.5 fM. It is important to underline that the double resonance allows to obtain a redundant measurement of the tested sample, hence it provides the sensor of an unprecedented, inherent and independent confirmation of the experimental results, giving rise to an interesting analytical advantage over the existing optical sensing solutions.

## Materials and methods

### Chemicals

All chemicals are listed in the Supplementary information and were used as received.

### SPR–LMR sensor fabrication

The fabrication process of the SPR–LMR platforms was as follows. A plastic optical fiber (POF) with a poly (methyl methacrylate) (PMMA) core of 980 μm diameter and a 10 μm diameter cladding (1 mm total diameter) was fixed by glue in a resin block that has a role of support. To obtain a D-shape sensitive area, the fiber was lapped by using two different types of polishing paper (1 and 5 μm grits), in such a way that the cladding and a part of the core were removed.

Titanium and Zirconium precursors solutions were prepared following the same protocol described in Stehlin et al.^45^. Firstly, the metal alkoxide precursor was mixed with methacrylic acid (MAA) and stirred for 5 min. Then, 2 mL n-propanol was added, and the solution was stirred for ten more minutes, before the addition of deionized water (DI). The formulations were prepared in order to respect molar ratios of Ti:MAA:DI = 1:8:20 and Zr:MAA:DI = 1:10:22, in order to prepare Ti-(TiOC) and Zr-oxo clusters (ZrOC) solutions, respectively. The resulting formulation was stirred for an extra hour and an ageing step of 24 h was required. In order to obtain a specific viscosity, the dilution rate was adjusted after these 24 h by adding n-propanol. A bilayer formed by Zirconium oxide (ZrO_2_) and Titanium oxide (TiO_2_) was deposited on the POF, according to the procedure described in ^30^. The key-step after spin-coating deposition of the precursor solution was a deep-UV laser irradiation performed with an excimer laser (Coherent/Excistar, 193 nm) that allows materials curing at room temperature. This process enabled the integration of high refractive index Metal Oxide thin film on plastic substrates that are not compatible with thermal annealing at temperatures such as 300–500 °C.

Two sensor configurations were explored: a first one, named “ZrO_2_–TiO_2_–Au configuration”, which consisted of a bilayer ZrO_2_–TiO_2_ formed by a ZrO_2_ nanolayer (thickness of about 40 ± 2 nm) deposited upon the exposed core of the fiber and a TiO_2_ layer (thickness of about 40 ± 2 nm) on the top of the latter; and a second one, named “TiO_2_–ZrO_2_–Au configuration”, which consisted of a bilayer TiO_2_–ZrO_2_, similar to the latter but obtained by swapping the position of the metal oxides. In both configurations, the total thickness of the metal oxide bilayer was equal to about 80 ± 2 nm. The final step, common to both configurations, was the deposition of a 60 nm gold film sputtered onto the metal-oxide bilayer by using a sputter coater machine (Safematic CCU-010, Zizers, Switzerland).

### Synthesis of soft nanoMIPs and functionalization of the sensing platforms

The synthesis of the nanoMIPs was according to Cennamo et al.^36^ The detailed synthetic protocol is reported in the Supplementary information. Selectivity was conferred to the SPR–LMR platforms by the covalent functionalization of the gold layer with nanoMIPs ^36^. A self-assembled monolayer (SAM) of carboxylic acids on the POF was obtained by an overnight derivatization with 300 μM (R)-α-lipoic acid in ethanol 8% v/v. After rising with water, the POF was incubated 1 h in 10 mM Lys-Lys, 50 mM EDC/NHS (1:1 mol:mol) in MES buffer 10 mM pH 5.5. NanoMIPs (10 μM) were re-suspended in MES buffer for 3 h under mild agitation and filtered on a 0.22 μm filter device prior to use. The nanoMIPs were added of 50 mM EDC and 50 mM NHS for a final volume of 0.6 mL. An aliquot of 60 μL was placed onto the POF let react for 2 h at room temperature in a sealed humid box. The nanoMIP-platforms were then washed extensively in 15 mL Falcon tubes (water 1 h; CHES buffer 10 mM pH 9.3 1 h, water 2 h) prior to use.

### Atomic force microscopy

Surfaces topography was studied using a NT-MDT Solver Pro system equipped with S7 scanner. Samples were imaged in semi-contact mode using silicon tips (NSG10_DLC, NT-MDT, 1 nm nominal tip radius, resonant frequency 255 Hz, force constant 11.5 N/m), collecting 1 × 1 µm, 512 points resolution topography images, acquired on different regions of each sample ^46^.

### Experimental setup

To test the presented configurations, a low-cost and simple setup has been used. More specifically, it consists of a white light source (HL–2000–LL, manufactured by Ocean Optics, Orlando, FL, USA) characterized by a wavelength emission range from 360 to 1700 nm used as a source and a spectrometer (FLAME-S-VIS-NIR-ES, Ocean Optics, Orlando, FL, USA) having a detection range between 350 and 1000 nm used as the receiver. The SPR–LMR platforms have been placed between the source and the receiver, and SMA connectors were used to connect all components of the described setup. The spectrometer was connected to a laptop in order to process the experimental data. Figure S1 (Supplementary Information) reports the picture of the experimental setup.

### Binding experiments

The platforms were tested for binding of their target analyte, HTR, by using a volume of 100 μL of analyte solution in the concentration range 17 fM–28 nM on the sensitive areas. After 5 min incubation, a PBS washing step was carried out and the spectra were acquired using the blank solution (PBS) as a bulk solution. The absolute value of the shift in resonance wavelength (SPR and LMR), calculated with respect to the blank (i.e. solution without the analyte), versus the different HTR concentrations were plotted in semi-log scale. Data were fitted with the Langmuir model Eq. (1) here reported:

1 $$\left|\Delta \lambda \right|=\left|{\lambda }_{c}-{\lambda }_{0}\right|=\left|\Delta {\lambda }_{max}\right|\cdot \left(\frac{c}{K+c}\right)$$

where ${\lambda }_{c}$ is the resonance wavelength at the analyte concentration *c*, ${\lambda }_{0}$ is the resonance wavelength value at the blank, $\Delta {\lambda }_{max}$ is the maximum value of Δλ (calculated by the saturation value minus the blank value) and *K* is a dissociation constant. The Langmuir equation model and the fitting were from OriginPro software (Origin Lab. Corp., Northampton, MA, United States).

## Supplementary Information

Supplementary Information.

## Data Availability

The datasets used and/or analysed during the current study available from the corresponding author on reasonable request.
