# Supplementary material for: Soft molecularly imprinted nanoparticles with simultaneous lossy mode and surface plasmon multi-resonances for femtomolar sensing of serum transferrin protein
Source: Sci Rep. 2023 Jul 11;13:11210. doi: 10.1038/s41598-023-38262-y (PMC10336098; doi:10.1038/s41598-023-38262-y)
Supplement: Supplementary file 1 — Supplementary Information. [file 41598_2023_38262_MOESM1_ESM.docx]

**Supplementary Information**

**Soft Molecularly Imprinted Nanoparticles with Simultaneous Lossy Mode and Surface Plasmon Multi-Resonances for Femtomolar Sensing of Serum Transferrin Protein**

Francesco Arcadio ^1^, Laurent Noël ^2,3^, Domenico Del Prete ^1^, Devid Maniglio ^4^, Mimimorena Seggio ^5^, Olivier Soppera ^2,3^, Nunzio Cennamo ^1^, Alessandra Maria Bossi ^5^*and Luigi Zeni ^1^*

^1^ University of Campania Luigi Vanvitelli, Department of Engineering, via Roma 29, 81031 Aversa, Italy;

^2^ University of Upper-Alsace, CNRS, IS2M UMR 7361, 68100 Mulhouse, France;

^3^ Université de Strasbourg, 67000 Strasbourg, France;

^4^ University of Trento, Department of Industrial Engineering, Via Sommarive 34, 38123, Trento, Italy;

^5^ University of Verona, Department of Biotechnology, Strada Le Grazie 15, 37134 Verona, Italy;

**List of contents**

1. **List of chemicals**
2. **Protocol for the synthesis of soft nanoMIPs**
3. **Experimental setup**
4. **Resonance wavelength analysis**
5. **Binding isotherms**
6. **List of chemicals**

Acrylamide (Aam), α-lipoic acid, 1-ethyl-3-(-3-dimethylaminopropyl) carbodiimide hydrochloride (EDC), N-hydroxysuccinimide (NHS), Lysil-lysine (Lys-Lys), methacrylic acid (MAA), N-tert-butylacrylamide (TBAm), N,N’-methylene bisacrylamide (BIS), N,N,N’,N’-tetramethyl ethylenediamine (TEMED), ammonium persulfate (APS), sodium dodecyl sulfate (SDS), N-Cyclohexyl-2-aminoethanesulfonic acid (CHES), Tris (hydroxymethyl)-aminomethane (TRIS), sodium dihydrogen phosphate, disodium monohydrogen phosphate, hydrochloric acid, sodium hydroxide, sodium chloride, Tween-20, acetonitrile, acetic acid, ethanol, were from Sigma-Aldrich (Darmstadt, Germany). The proteins: human serum transferrin (HTR), proteinase-k, microperoxidase, cytochrome c, human serum albumin, trypsin were from Sigma-Aldrich (Darmstadt, Germany). A 40% w/w APS stock solution was freshly prepared prior to polymerization. Titanium (IV) tetraisopropoxide (97%) and zirconium (IV) tetrapropoxide (70% in n-propanol) were purchased from Merck and used as received. Methacrylic acid (99%) was also purchased from Merck, and n-propanol (99%) from Alfa Aesar.

1. **Protocol for the synthesis of soft nanoMIPs**

A total monomer concentration of 0.1% (w/v) (7.2 mM) was used. Acrylamide (Aam), methacrylic acid (MAA), N-tert-butylacrylamide (TBAm) were added at 8, 8 and 4% (mol/mol) respectively, together with 80% (mol/mol) of N,N’-methylenebisacrylamide (BIS) in 20 mM phosphate buffer (PB) pH 7.4. The solutions were filtered with a 0.22 μm filter. The template, human serum transferrin (HTR), was added to the MIP-vials to the final concentration of 1.2 μM. Vials were closed with rubber caps, sonicated for 10 min and bubbled with N2 for 30 min. Then APS (0.04% w/v) and TEMED (0.03% w/v) were added and the polymerization was carried out at 20 °C for 20 h. At the end of the polymerization, the template was removed by the addition of Trypsin at 1:25 (w/w) ratio with respect to the template, and incubated for 2 h at 30 °C. The nanoparticles (nanoMIPs) were then washed against 3 L of MilliQ water using a Vivaflow 50 system (100,000 MWCO) (Sartorius Stedim, Firenze, Italy). The yield of polymerization was calculated from the weight of the lyophilized nanoMIPs with respect to the total weight of the monomers added to the synthetic batch.

1. **Experimental setup**


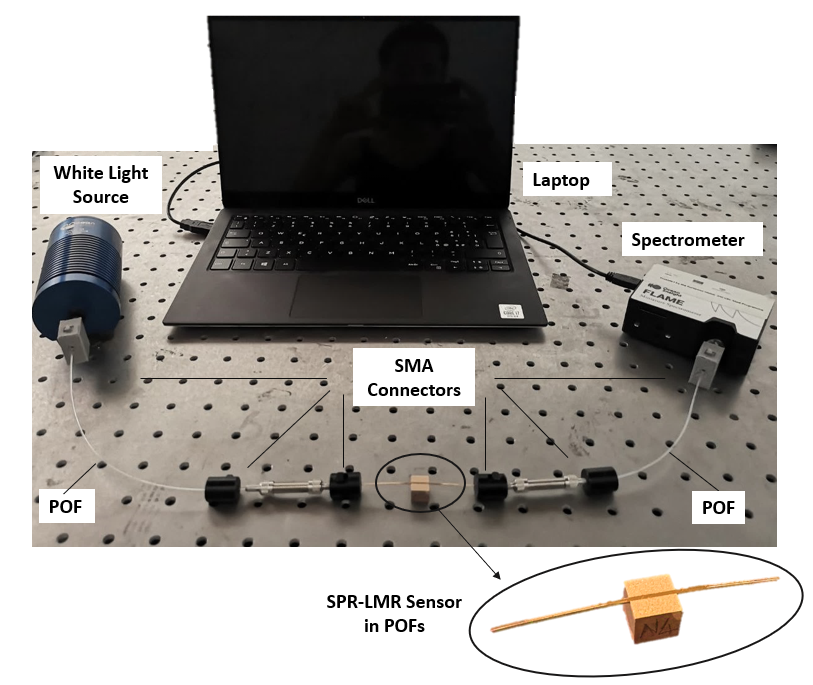


**Fig S1:** Picture of experimental setup used to test the SPR-LMR platforms.

1. **Resonance wavelength analysis**


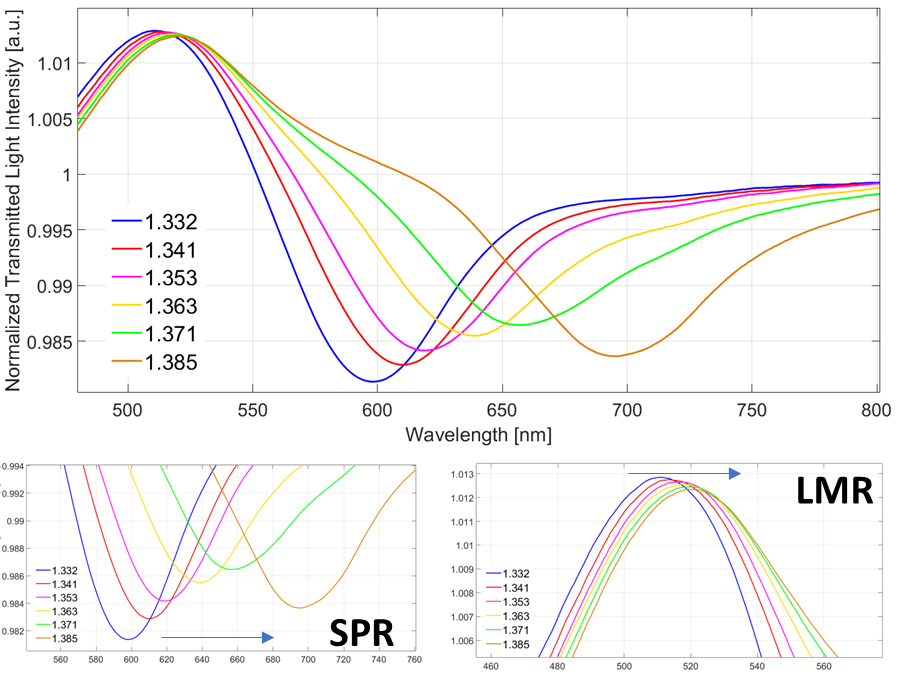


**Figure S2** ZrO_2_-TiO_2_-Au optical characterization: normalized spectra obtained before the nanoMIP functionalization at different external refractive indices and enlargement of the two resonance wavelength areas. The spectra obtained are normalized to the spectra in air (i.e., where the resonance condition is not satisfied).


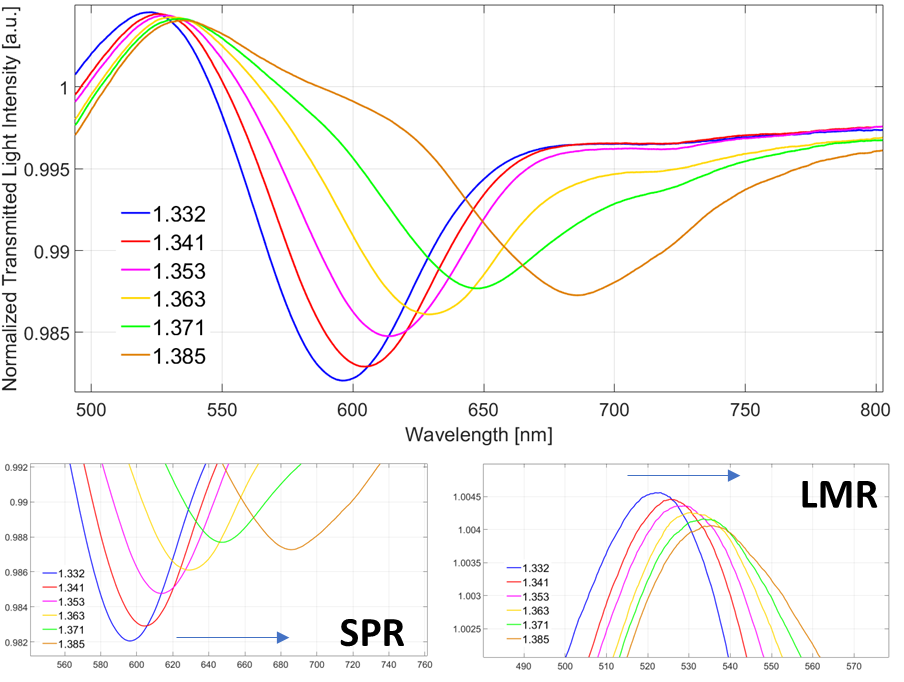


**Figure S3** TiO_2_-ZrO_2_-Au optical characterization: normalized spectra obtained before the nanoMIP functionalization at different external refractive indices and enlargement of the two resonance wavelength areas. The spectra obtained are normalized to the spectra in air (i.e., where the resonance condition is not satisfied).


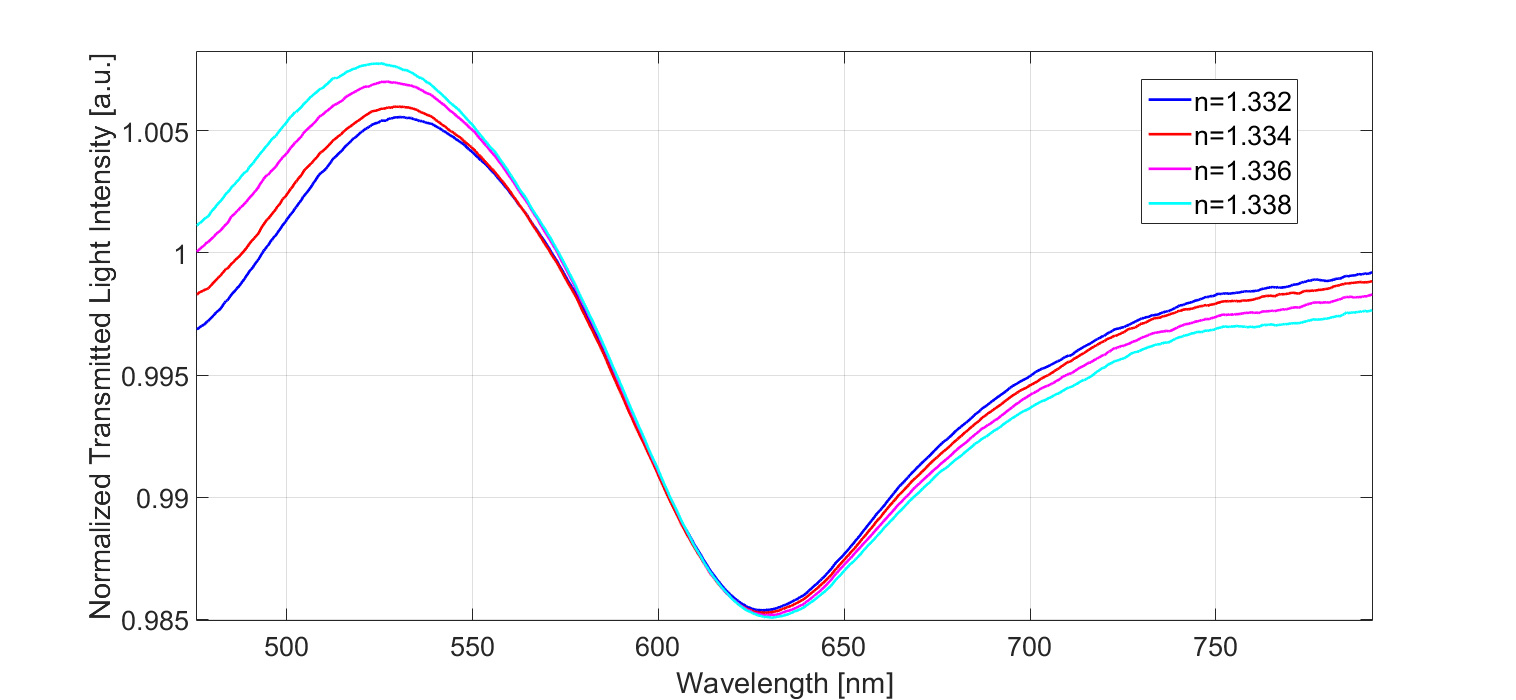


**Figure S4** ZrO_2_-TiO_2_-Au-nanoMIP optical characterization: normalized spectra obtained after the nanoMIP functionalization at different external refractive indices and enlargement of the resonance wavelength areas.


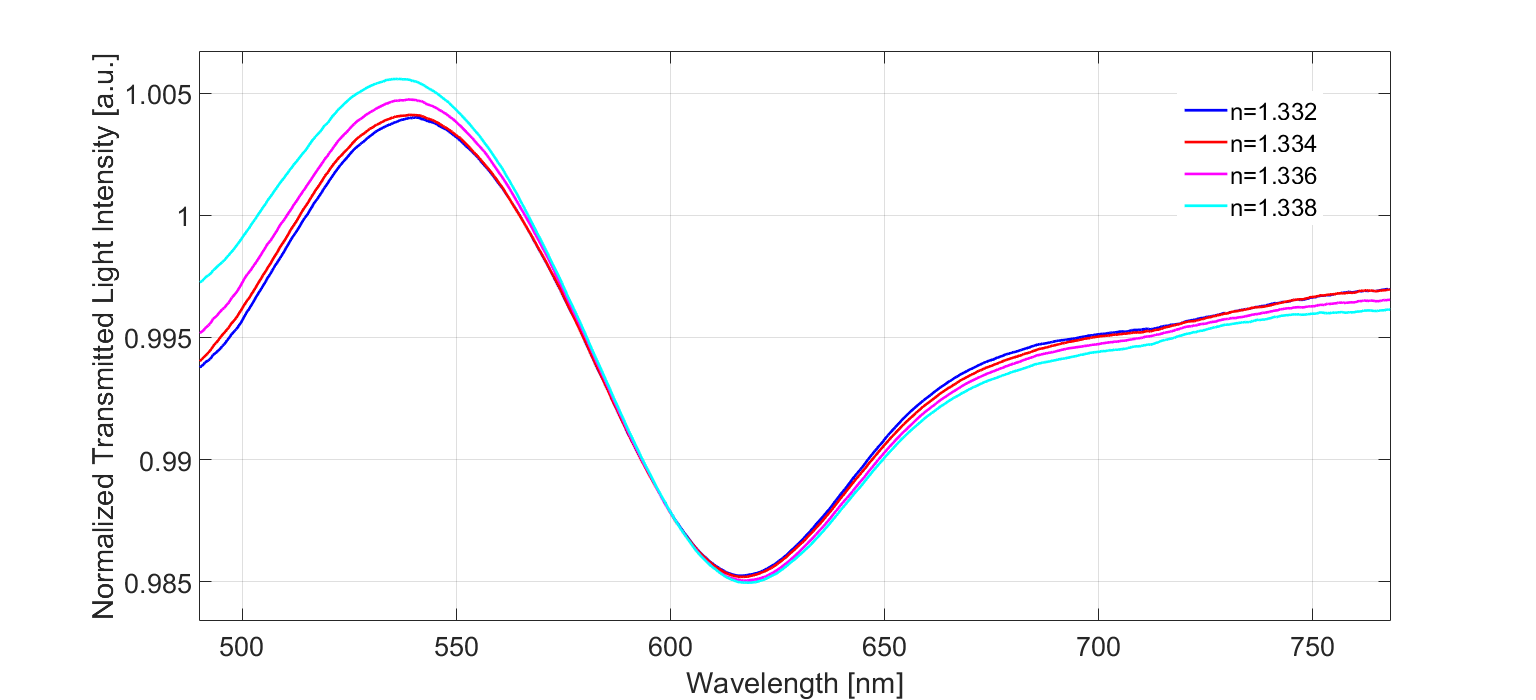


|  |  |
| --- | --- |

**Figure S5** TiO_2_-ZrO2-Au-nanoMIP optical characterization: normalized spectra obtained after the nanoMIP functionalization at different external refractive indices.

1. **Binding isotherms**

*
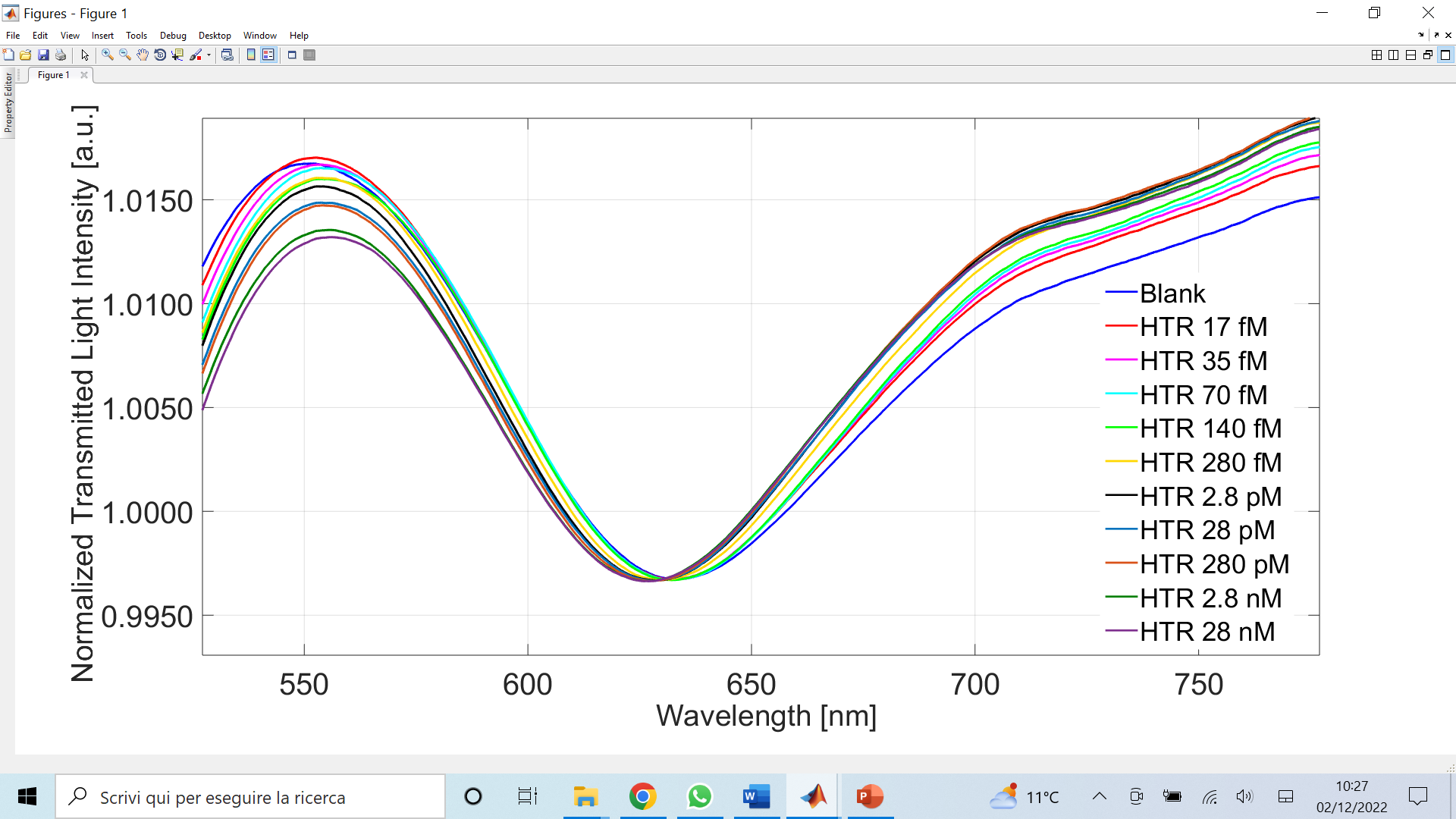
*

**Fig S6:** Normalized transmitted spectra acquired at different HTR concentrations for ZrO_2_-TiO_2_-Au-nanoMIP.

**Table S1.** Langmuir fitting parameters for both SPR and LMR peaks upon HTR binding on ZrO_2_-TiO_2_-Au-nanoMIP configuration.

| **Resonance** | **λ_0_**  **[nm]** | **Δλ_max_**  **[nm]** | **K**  **[fM]** | **Statistics** | |
| --- | --- | --- | --- | --- | --- |
|  |  |  |  | **χ^2^** | **R^2^** |
| SPR | 0.17 ± 0.5 | 5.87 ± 0.3 | 54 ± 37 | 3.237 | 0.935 |
| LMR | 0.87 ± 0.7 | 5.54 ± 0.2 | 31 ± 27 | 9.466 | 0.908 |


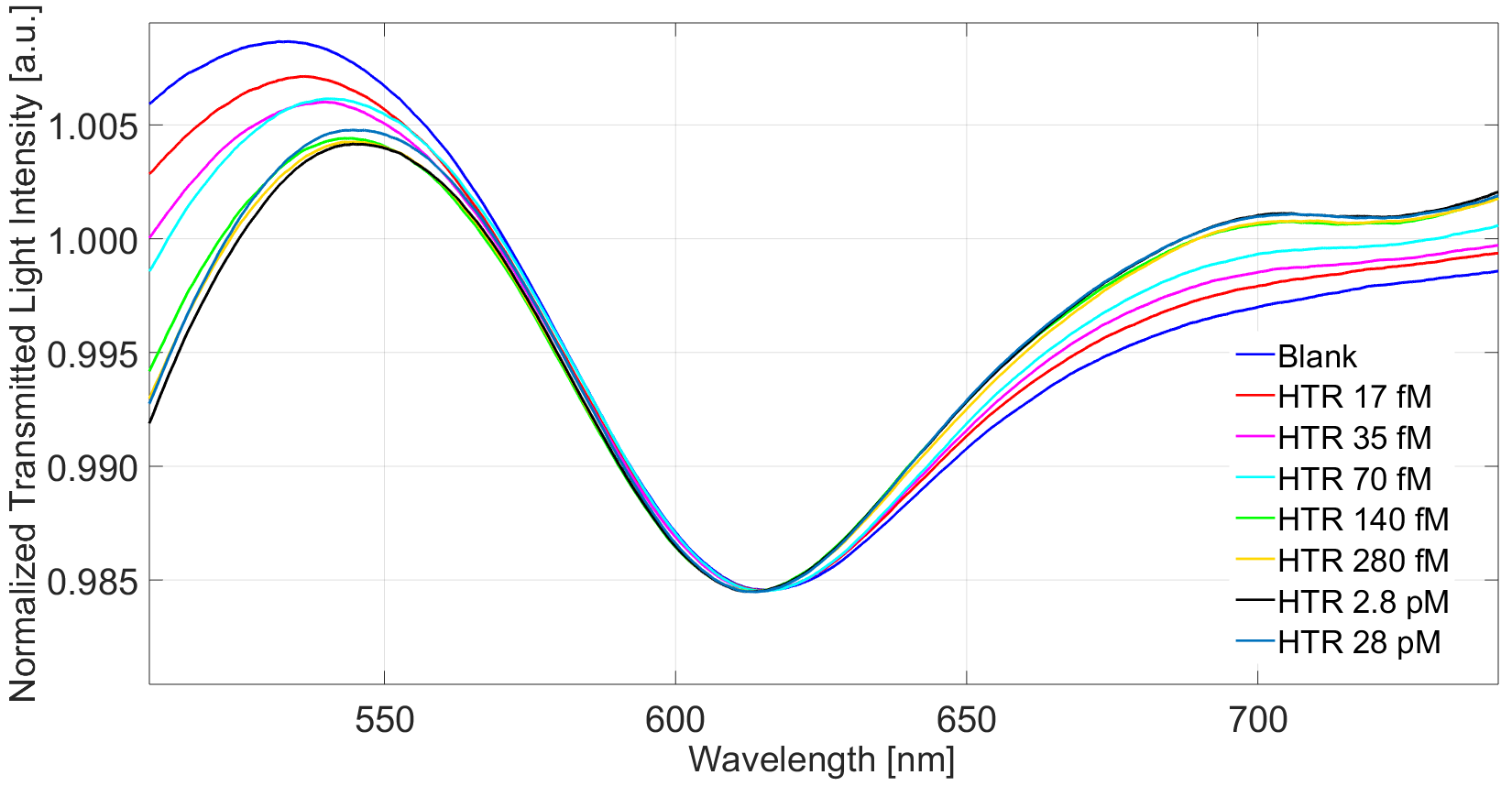


**Fig S7:** Normalized transmitted spectra acquired at different HTR concentrations for TiO_2_-ZrO_2_-Au-nanoMIP.

**Table S2.** Langmuir fitting parameters for both SPR and LMR peaks upon HTR binding on TiO_2_-ZrO_2_-Au-nanoMIP configuration.

| **Resonance** | **λ_0_**  **[nm]** | **Δλ_max_**  **[nm]** | **K**  **[fM]** | **Statistics** | |
| --- | --- | --- | --- | --- | --- |
|  |  |  |  | **χ^2^** | **R^2^** |
| SPR | -0.37 ± 0.3 | 3.28 ± 0.2 | 53 ± 32 | 1.875 | 0.955 |
| LMR | -0.36 ± 1.2 | 12.75 ± 0.5 | 32 ± 12 | 9.367 | 0.985 |
